# Oophoropexy to the Round Ligament after Recurrent Adnexal Torsion

**DOI:** 10.1055/s-0038-1673426

**Published:** 2018-10-11

**Authors:** Inês Sarmento Gonçalves, Joana Sampaio, Joana Félix, Ana Catarina Silva, Gisela Fornelos, Pedro Tiago Silva

**Affiliations:** 1 Department of Obstetrics and Gynecology, Unidade Local de Saúde de Matosinhos, Matosinhos, Portugal; 2 Department of Radiology, Unidade Local de Saúde de Matosinhos, Matosinhos, Portugal

**Keywords:** Adnexal Torsion, oophoropexy, laparoscopy, round ligament, gynecological emergency, torção anexial, ooforopexia, laparoscopia, ligamento redondo, emergência ginecológica

## Abstract

Recurrent adnexal torsion is a rare gynecological emergency. We report a case of recurrent ipsilateral adnexal torsion in a woman with polycystic ovaries, previously submitted to a laparoscopic plication of the utero-ovarian ligament. Due to the recurrence after the plication of the utero-ovarian ligament, the authors performed a laparoscopic oophoropexy to the round ligament, which is an underreported procedure. The patient was asymptomatic for 1 year, after which she had a new recurrence and needed a unilateral laparoscopic adnexectomy. Since then, she regained the quality of life without any gynecological symptoms.

Oophoropexy to the round ligament may be considered when other techniques fail or, perhaps, as a first option in selected cases of adnexal torsion, as it may allow the prevention of recurrence without increasing morbidity while preserving the adnexa.

## Abstract (pt)

**Resumo:**

A torção anexial recorrente é uma emergência ginecológica rara. Os autores descrevem um caso de torsão anexial unilateral recorrente em uma paciente com síndrome de ovário policístico, previamente submetida a plicatura do ligamento utero-ovárico por laparoscopia. Nesta circunstância, os autores decidiram realizar uma ooforopexia laparoscópica ao ligamento redondo, uma técnica pouco descrita na literatura. A paciente manteve-se assintomática durante 1 ano, período após o qual teve nova recorrência, tendo-se decidido realizar uma anexectomia laparoscópica unilateral. Desde então, ela recuperou a qualidade de vida sem qualquer sintoma ginecológico.

A ooforopexia ao ligamento redondo é uma técnica que deverá ser considerada quando outras falham e, em casos selecionados de torsão anexial recorrente, poderá ser considerada a primeira abordagem, para prevenir a recorrência e preservar o anexo.

## Introduction

Adnexal torsion, although rare, accounts for 2.5 to 5% of all gynecological emergencies.^1^ Its diagnosis may be challenging because of poorly defined clinical findings, and the consequences of a missed diagnosis can be devastating, as prolonged torsion can lead to ovarian atrophy and premature menopause.^1^ ^2^ Recurrent ovarian torsion is much rarer, but, although easier to diagnose, its treatment poses a great challenge.^3^ We report a rare case of recurrent adnexal torsion after plication of the utero-ovarian ligament apparently successfully treated with a laparoscopic oophoropexy to the round ligament, an underreported procedure. Unfortunately, the ovarian torsion recurred after 1 year of follow-up and a unilateral adnexectomy was performed.

## Case Presentation

**Fig. 1:**
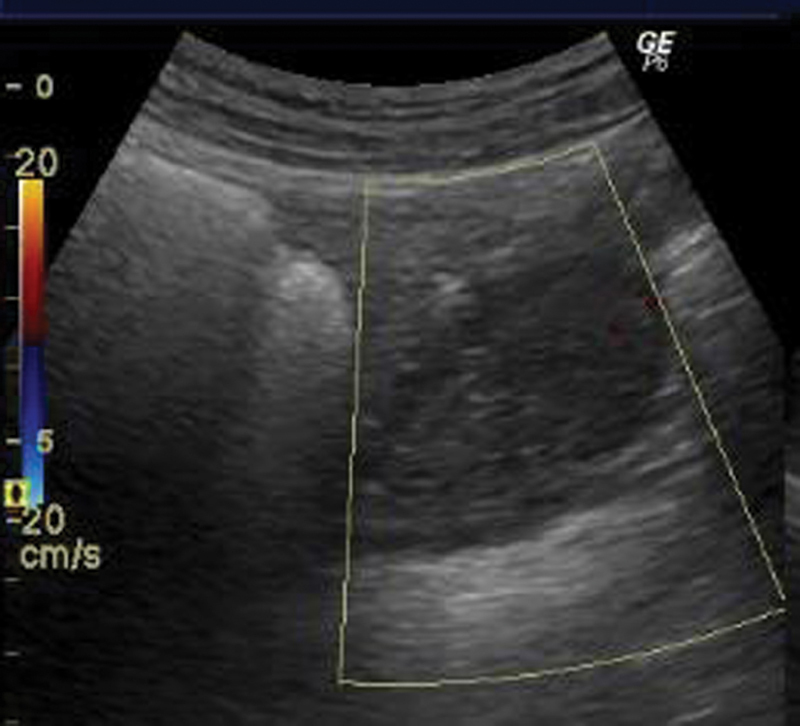
Ultrasound of the right ovary: multiple small follicles in the periphery and no Doppler color sign.

A 31-year-old woman with an uneventful medical history, except for polycystic ovarian syndrome manifested by bilateral polycystic ovaries and oligomenorrhea, without any other medication besides combined oral contraceptive pills, presented to the gynecology emergency room with sudden right pelvic pain. Findings at the clinical examination and sonography (Fig. 1) were suggestive of acute right adnexal torsion.

**Fig. 2:**
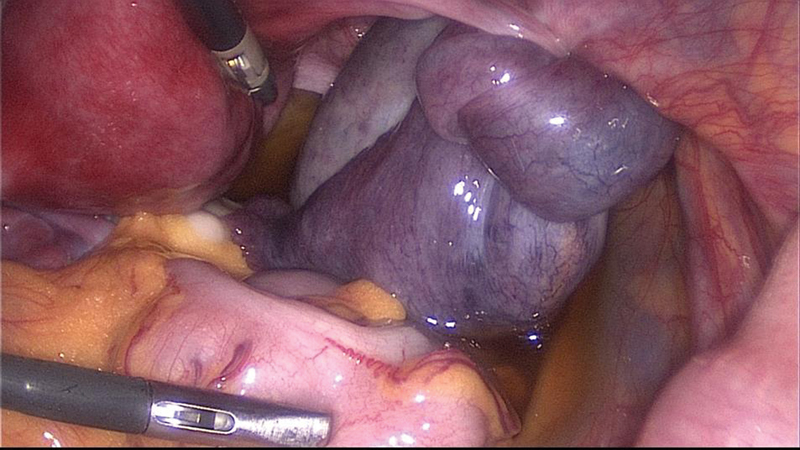
Laparoscopy performed during the first episode of recurrent adnexal torsion: obvious torsion and swelling of the right enlarged adnexa.

**Fig. 3:**
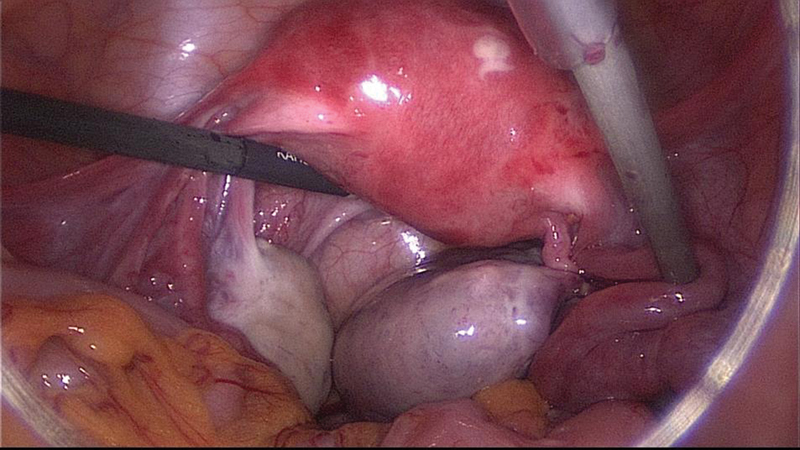
Laparoscopy performed during the first episode of recurrent adnexal torsion: plication of the utero-ovarian ligament with a non-absorbable suture.

A laparoscopy was performed confirming the torsion of an enlarged polycystic right ovary. The detorsion was accomplished and the purplish right ovary and tube regained their normal color. Three months later, the patient returned to our emergency room with similar symptoms and, once more, the findings were suggestive of recurrent torsion of the right adnexa. Another laparoscopic exploration confirmed the diagnosis (Fig. 2) and a plication of the utero-ovarian ligament with a non-absorbable suture was performed (Fig. 3).

**Fig. 4:**
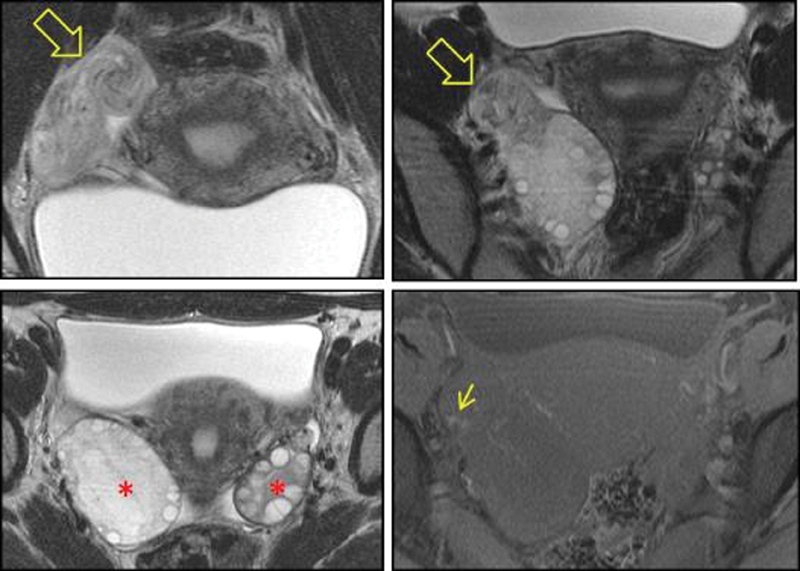
Magnetic resonance imaging evaluation: Coronal and Axial T2WI nicely demonstrates the twisting of the ovarian pedicle - the whirlpool sign (open arrow); the enlargement of the right ovary compared with the left one is also evident (asterisk); Fat Sat T1WI shows the presence of blood in the vascular pedicle in favor of vascular congestion (small arrow).

Two months after the second surgery, the patient started experiencing a milder constant right abdominal discomfort that worsened with physical activity, affecting her professional activities for 2 weeks. The vaginal Doppler ultrasound was inconclusive, and a magnetic resonance imaging (MRI) was performed, which revealed an enlarged edematous right ovary with ipsilateral abnormal ovarian enhancement, suggestive of a subacute process (Fig. 4).

**Fig. 5:**
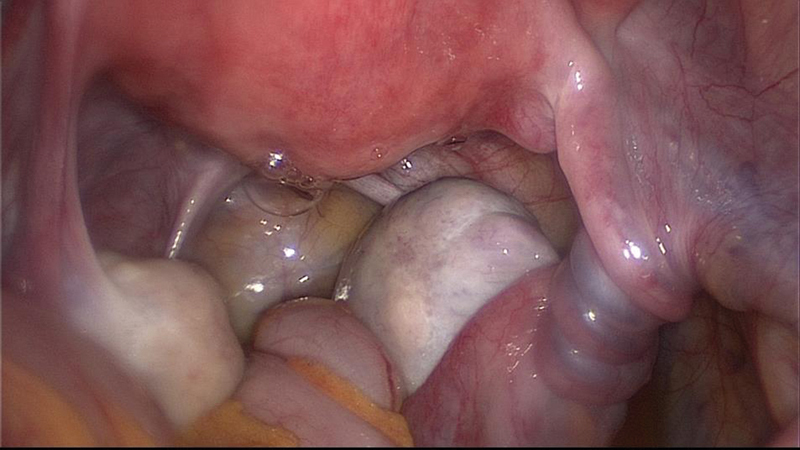
Laparoscopy performed during the second episode of recurrent right adnexal torsion, 2 months after plication of the utero-ovarian ligament.

Another laparoscopy confirmed the second recurrence of right adnexal torsion (Fig. 5).

**Fig. 6:**
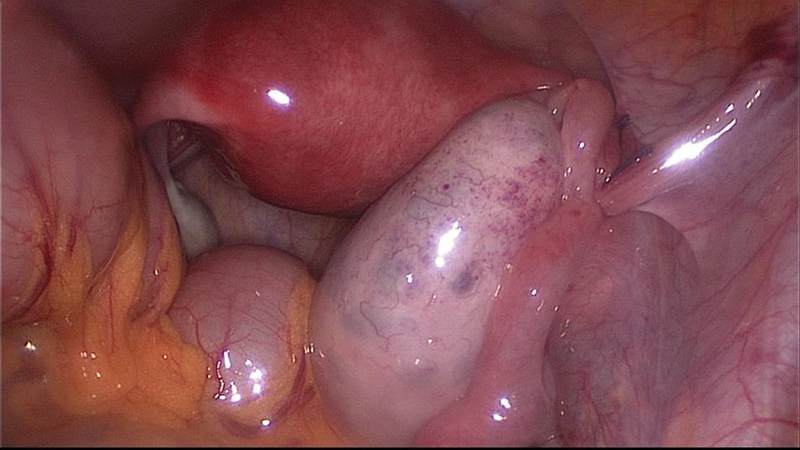
Laparoscopy performed during the second episode of recurrent right adnexal torsion: fixation of the right adnexa to the ipsilateral round ligament with two interrupted sutures, through the mesosalpinx.

A gentle and careful detorsion was performed and both the ovary and the tube appeared healthy and well perfused, with a disrupted right utero-ovarian ligament in the same location of the previous plication. Although the pelvis appeared normal, subjectively it seemed narrow, and the tube was thin and elongated with a lax mesosalpinx. A different oophoropexy was executed, consisting of two fixation points between the right adnexa and the ipsilateral round ligament: one stitch fixing the medial and the lateral parts of the disrupted utero-ovarian ligament to the round ligament, and another stitch from the right ovarian capsule near the ovarian hilum to the round ligament. The two fixation stitches were performed through the mesosalpinx, below the right tube, with non-absorbable sutures. After the first stitch, the surgeons realized that the ovary was still very mobile, hence the need for the second fixation point, bringing the ovary closer to the uterus and, at the same time, decreasing its mobility (Fig. 6).

The postoperative period was uneventful, and the patient was discharged the next day. A vaginal ultrasound was performed 2 months after the procedure and demonstrated normally located ovaries with cortical follicles and arterial and venous normal blood flow and similar volumes. The patient completed 1 year of follow-up without any relevant symptom, after which the right adnexa torsion recurred. After careful explanation and informed patient consent, we opted to perform a unilateral right adnexectomy. The surgery was uneventful with a normal postoperative period. The anatomopathological examination revealed a necrotic and hemorrhagic ovary. The patient is currently completing 2 years of follow-up and regained her quality of life without any symptoms of contralateral ovarian torsion recurrence.

## Discussion

Adnexal torsion is a gynecologic emergency.^4^ The majority of cases occur due to adnexal enlargement (ovarian or paraovarian cysts, polycystic or hyperstimulated ovaries), preventing the return to the natural position after adnexal twisting.^2^ ^5^ Fortunately, the majority of cases of polycystic ovaries or ovarian cysts do not complicate with an adnexal torsion, meaning that there should be other reasons for this condition.^6^ In addition, torsion can occur in normal-appearing ovaries.^7^ Some authors suggest that hyperlax mesosalpinx or meso-ovarium, with or without connective tissue disorders, may also play an important role.^6^

In our patient, polycystic ovaries have predisposed to the adnexal torsion. However, the ipsilateral recurrence suggests other possible underling factors. The authors outline the hyperlax meso-ovarium in a narrow pelvis as being a possible anatomic predisposing factor in this case.

Laparoscopy is important for a prompt diagnosis, and surgical intervention at the beginning of the clinical presentation is essential for adnexal preservation.^8^ In fact, a delay of 36 hours in the intervention results in significant congestion and necrosis.^7^ However, the presentation of adnexal torsion is nonspecific and the definitive diagnosis is based upon surgical findings.^7^ Doppler ultrasound may aid in the diagnosis of adnexal torsion, but it fails to recognize ∼ 60% of the surgically diagnosed ovarian torsion cases.^9^ Recurrent torsion is easier to diagnose because of the familiarity of the patient with the symptoms and of the higher suspicion index.^3^ In this case, a Doppler vaginal sonography confirmed the clinical suspicion in the first two episodes of adnexal torsion but failed to recognize the third. Therefore, the presence of normal blood flow in the vaginal ultrasound should not exclude the diagnosis of torsion, whereas the absence of Doppler flow is highly specific but not too sensitive.^9^ An MRI exam can help in the diagnosis and in the etiology of adnexal torsion, especially when the ultrasonographic evaluation is doubtful, as occurred in the second recurrence in our patient.

Manhes et al^10^ first described the importance of laparoscopy in adnexal torsion cases, offering an opportunity for the adnexa to be untwisted (allowing ischemic adnexa to regain follicular activity), to estimate the severity of the adnexal damage, to elucidate the etiology and to proceed to specific laparoscopic surgical treatment. Fortunately, since then, adnexectomy has been largely abandoned and substituted by this laparoscopic conservative management.^4^ ^8^ In fact, the ovarian function is preserved despite a necrotic appearance in > 90% of the ovaries that are detorsed.^7^ However, conservative management may predispose to recurrence.^4^ Surgical management to prevent recurrence has included the plication of the utero-ovarian ligament and several forms of oophoropexy involving fixation of the ovary to the posterior aspect of the uterus, to the round ligament, to the ovarian fossa, or to the lateral pelvic wall.^3^ ^4^ Several techniques have been proposed and this prophylactic surgery is increasingly advocated by many authors, but there is still a considerable controversy regarding the indications, the timing, and the preferred surgical method.^4^

In our patient, the first prophylactic surgery was only performed at her second torsion event. Although the authors are aware of the higher risk of torsion recurrence because it occurred in an enlarged ovary due to a polycystic ovarian syndrome, the decision of performing a simple adnexal detorsion was based on the fact that prophylactic adnexal surgery is not without risks, as it may interfere with tubal blood supply, tubal function, or with ovarian communication with the fallopian tube.^4^ The first prophylactic adnexal surgery performed in our patient was the plication of the ipsilateral utero-ovarian ligament. The authors have performed this same procedure in other cases of recurrent adnexal torsion with good outcomes, and this was their first case of recurrence after utero-ovarian ligament plication. This oophoropexy is the preferred method of several authors, for the time being, because it supposedly has less effect on future fertility with minimal interference with the normal anatomy, is relatively easy to perform, and does not increase the potential for immediate postoperative adverse outcome.^4^ However, when the patient presented with the second recurrence episode, the authors assumed that the normal length utero-ovarian ligament plication had failed to prevent the recurrence of adnexal torsion and opted for a different approach.

The alternative conservative prophylactic approaches include suturing the ovary to the pelvic sidewall.^1^ ^4^ Sheizaf et al^3^ recently described a repeated adnexal torsion after a utero-ovarian ligament plication in a prepubertal girl successfully treated with ovarian fixation to the ipsilateral pelvic wall. However, some authors claim that this method might cause excessive tension on the adnexa and impede the function of the fallopian tube.^4^ There are only two published cases of successful ovary fixation with plication to the round ligament after recurrent ipsilateral ovarian torsion.^4^ ^8^ We have opted to perform a laparoscopic oophoropexy to the ipsilateral round ligament with two anchoring sutures affixed to the medial and lateral ends of the disrupted right utero-ovarian ligament and to the ovarian capsule near the hilum to restore the normal anatomy, stabilizing both the ovary and the tube without harming the ovarian function or causing chronic pelvic pain. To the best of our knowledge, this is the first published case of recurrent adnexal torsion treated with this new technique, fixing both the medial and the lateral axis of the ovary to the round ligament. Yet, it remains unclear whether this surgical procedure will have some impact in terms of fertility, since this was not tested.

Our patient completed 1 year of follow-up without any symptoms, and we were convinced that we had solved her problem. However, 1 year after oophoropexy to the round ligament, the right adnexal torsion recurred, and, after careful medical discussion and patient consent, we opted to perform a unilateral adnexectomy. We believe that, in this case, the main reason for the adnexal torsion recurrence is due to an anatomical local feature rather than to a systemic cause.

The authors presented an uncommon case report of adnexal torsion recurrence after a utero-ovarian plication. To prevent another recurrence, an oophoropexy with fixation to the ipsilateral round ligament was performed, decreasing the ovarian mobility. This is an underreported technique to consider when others fail or, perhaps, as a first option in selected cases, as it may allow the prevention of recurrence without increasing morbidity while preserving the adnexa. However, we must bear in mind that adnexal torsion recurrence is a possible early or late complication after all kinds of oophoropexies and that adnexectomy is an adequate alternative option in some cases, allowing the patient to regain her quality of life.
